# Development of a point-of-care dual one-step recombinase-aided PCR assay for rapid identification of *Mycobacterium tuberculosis gyrA* mutations conferring fluoroquinolone resistance

**DOI:** 10.3389/fmicb.2026.1772984

**Published:** 2026-03-02

**Authors:** Xingyu Liu, Kenan Peng, Yuanrui Li, Shihao Jiao, Jianing Wu, Duoxiao Zhang, Shijue Gao, Yujie Xiang, Junkai Ren, Qian Ma, Xinxin Li, Zijin Zhao, Zhiqiang Han, Xinxin Shen, Xuejun Ma, Yanqing Tie

**Affiliations:** 1 Hebei Medical University, Shijiazhuang, Hebei, China; 2 Department of Clinical Laboratory, Hebei General Hospital, Shijiazhuang, Hebei, China; 3 Hebei Key Laboratory of Molecular Medicine, Shijiazhuang, Hebei, China; 4 Hebei Clinical Research Center for Laboratory Medicine, Shijiazhuang, Hebei, China; 5 National Key Laboratory of Intelligent Tracking and Forecasting for Infectious Diseases, NHC Key Laboratory of Medical Virology and Viral Diseases, National Institute for Viral Disease Control and Prevention, Chinese Center for Disease Control and Prevention, Beijing, China; 6 Health Science Center, Ningbo University, Ningbo, Zhejiang, China; 7 North China University of Science and Technology, Tangshan, Hebei, China; 8 Hebei North University, Zhangjiakou, Hebei, China

**Keywords:** fluoroquinolone resistance, *gyrA*, molecular diagnostics, *Mycobacterium tuberculosis*, point-of-care testing, recombinase-aided PCR

## Abstract

**Background:**

Fluoroquinolone (FQ) resistance in *Mycobacterium tuberculosis* (MTB) is a major cause of treatment failure in multidrug-resistant tuberculosis (MDR-TB). This resistance primarily results from mutations within the quinolone resistance-determining region (QRDR) of the *gyrA* gene encoding DNA gyrase. Conventional phenotypic drug susceptibility testing (DST) is labor-intensive and time-consuming, making it unsuitable for rapid clinical decision-making. Therefore, developing a rapid, sensitive, and point-of-care testing (POCT) assay is of great importance.

**Methods:**

A cartridge-based POCT dual one-step recombinase-aided PCR (POCT-DO-RAP) assay was established for rapid detection of FQ resistance-associated mutations in MTB. Locked nucleic acid (LNA) probes were designed to enhance single-nucleotide discrimination for *gyrA* A90V and D94G mutations. Magnetic bead-based extraction enabled fully automated nucleic acid purification, while recombinase-aided amplification (RAA) and quantitative PCR (qPCR) were sequentially performed within a real-time PCR-based POCT device. The analytical performance of the POCT-DO-RAP assay was evaluated using recombinant plasmids (1–105 copies/μL), H37Rv-simulated sputum samples and 128 clinical isolates. The POCT-DO-RAP assay was further validated using 88 clinical samples and the results were compared with the conventional qPCR and the nested PCR followed by Sanger sequencing.

**Results:**

The optimized POCT-DO-RAP assay achieved limits of detection of 1 copy/reaction for the wild-type (WT) tube and 10 CFU/mL for the mutant-type (MT) tube, representing a 10-fold increase in sensitivity compared with conventional qPCR. The assay reliably detected mutant alleles even when they represented only 1% of mixed templates. Among 128 clinical isolates, the assay accurately differentiated 50 wild-type and 78 resistant strains, showing complete concordance with Sanger sequencing and no cross-reactivity. In clinical validation,9 samples negative by qPCR were confirmed as positive by both DO-RAP assay and nested PCR followed by Sanger sequencing.

**Conclusion:**

The POCT-DO-RAP assay developed in this study achieves a fully integrated “sample-in, result-out” workflow on a single device, offering ultra-high sensitivity, precise mutation discrimination, and excellent clinical concordance. This approach provides a promising molecular diagnostic tool for rapid detection of drug-resistant tuberculosis, particularly suitable for primary healthcare and resource-limited settings.

## 1 Introduction

Tuberculosis (TB) remains one of the world’s most devastating infectious diseases. In 2023, TB likely surpassed COVID-19 as the leading cause of death from a single infectious agent, with an annual mortality rate nearly twice that of HIV/AIDS (World Health Organization [WHO], 2024). Globally, more than 10 million people develop TB each year, and this number has continued to rise since 2021. It is estimated that approximately one quarter of the world’s population is infected with *Mycobacterium tuberculosis* (World Health Organization [WHO], 2024).

Amid the increasing global TB burden, drug-resistant tuberculosis—particularly multidrug-resistant (MDR) and rifampicin-resistant TB (RR-TB)—has become a major obstacle to TB control. Each year, around 400,000 people develop MDR/RR-TB, with an incidence of 3.2% among new cases and as high as 16% among previously treated patients (World Health Organization [WHO], 2024). Both MDR-TB and RR-TB require treatment with second-line drugs, among which fluoroquinolones (FQs)—especially levofloxacin and moxifloxacin—are key components that largely determine therapeutic outcomes (He et al., 2025; World Health Organization [WHO], 2022). However, widespread FQ use in bacterial infections has led to the emergence of FQ-resistant strains. A significant proportion of MDR/RR-TB cases have evolved into pre-extensively drug-resistant (pre-XDR) TB with additional fluoroquinolone resistance, posing a severe threat to global TB elimination efforts (Nehru et al., 2024). Determining FQ resistance before treatment is crucial for guiding appropriate drug selection, avoiding ineffective regimens, and improving treatment success rates (Kim et al., 2025; Zhang et al., 2025). Therefore, rapid and accurate detection of FQ resistance is essential for optimizing MDR-TB therapy.

Traditional phenotypic drug susceptibility testing (DST) is time-consuming, technically demanding, and poses biosafety risks, limiting its utility for timely clinical decision-making. As a result, molecular diagnostic methods targeting drug-resistance-associated mutations have gained prominence. In TB, FQ resistance primarily results from mutations within the quinolone resistance-determining region (QRDR) of the *gyrA* gene (Chien et al., 2016; Uddin et al., 2021; Willby et al., 2020), particularly at codons 90 and 94, which together account for 55–95% of all FQ-resistant cases (Castro et al., 2020; Dixit et al., 2023; Zhou et al., 2025). Although mutations in *gyrB* are also associated with resistance, they occur less frequently and usually accompany *gyrA* mutations (Chaoui et al., 2018; Uddin et al., 2021).

Currently, several molecular assays are available for detecting drug resistance. Line probe assays (LPAs) cover key mutation sites but require complex manual operations and designated laboratory infrastructure (Havlicek et al., 2018). The automated Xpert MTB/XDR system simplifies testing but may lack the sensitivity required to detect low-abundance heteroresistant subpopulations (Chen et al., 2023). Given these limitations, there is a pressing need for a simplified, highly sensitive POCT assay that can be deployed in resource-limited settings.

A novel recombinase-aided PCR (RAP) method was previously developed that enables rapid and highly sensitive detection of nucleic acids (Fan et al., 2023, 2021), and further established a dual one-step RAP (DO-RAP) technique for detecting MTB isoniazid resistance genes, achieving a limit of detection as low as 2 copies per reaction and a heteroresistance detection sensitivity of 5% (Han et al., 2025). The principle of one-step RAP is to combine recombinase-aided amplification (RAA) and polymerase chain reaction (PCR) sequentially within a single buffer system: RAA initiates primary amplification, followed by qPCR-based secondary amplification with specific primers and probes. Compared with conventional qPCR, this approach provides markedly, improved sensitivity. However, broader clinical implementation—especially in resource-limited settings—requires further simplification and integration of nucleic acid extraction with the nucleic acid amplification.

**FIGURE 1:**
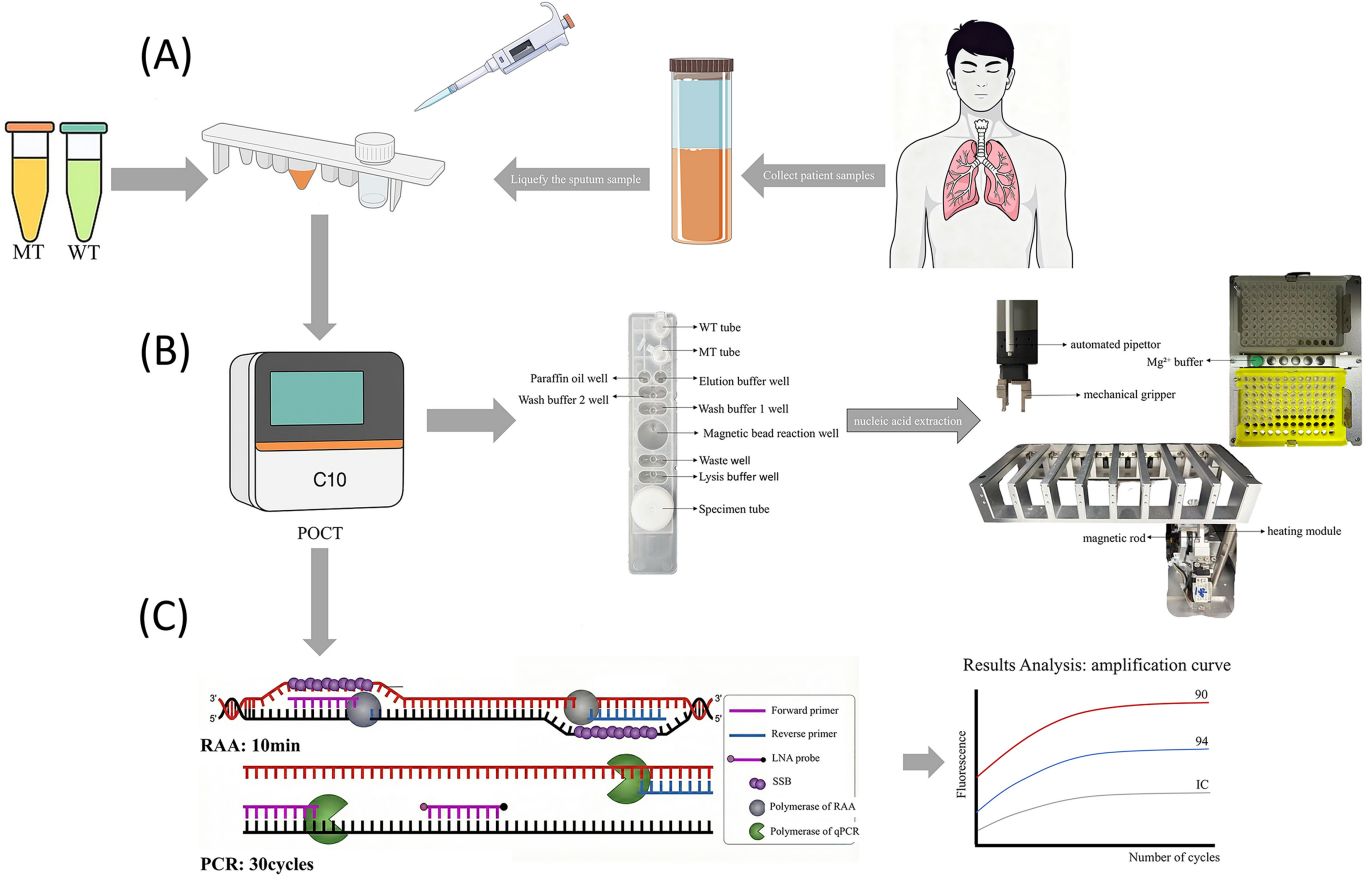
Operational workflow and principle of the POCT-DO-RAP system. **(A)** Sputum liquefaction and the separate loading of sample, WT mix, and MT mix into the cartridge. **(B)** Design of automated extraction and dispensing. **(C)** RAP Amplification and results.

In this study, the nucleic acid extraction process and reaction conditions were optimized to overcome the operational complexity of RAP detection. By integrating the DO-RAP method into a POCT device, a fully automated cartridge-based “sample-in, result-out” system was established. Building on this device, A POCT-based DO-RAP assay (POCT-DO-RAP) was developed targeting the major *gyrA* A90V and D94G mutations responsible for FQ resistance in MTB, with *IS1081* included as an internal control. Each cartridge contains two reaction tubes: a wild-type (WT) tube for detecting *gyrA*90 and *gyrA*94 wild-type sequences, and a mutant-type (MT) tube for detecting the A90V and D94G mutations. After the sample loading, the nucleic acid is automatically extracted and, evenly distributed into both reaction tubes, followed by the one-step RAP reaction, enabling rapid and sensitive detection of FQ resistance in MTB (Figure 1).

## 2 Materials and methods

### 2.1 Strains and specimens

A total of 128 MTB clinical isolates were included in this study, comprising both FQ-susceptible and FQ-resistant strains. In addition, five common respiratory bacterial isolates—*Haemophilus influenzae*, *Streptococcus pneumoniae*, *Staphylococcus aureus*, *Klebsiella pneumoniae*, and *Pseudomonas aeruginosa*—were used to assess assay specificity. All isolates were obtained from the Chinese Center for Disease Control and Prevention (China CDC), National Institute for Communicable Disease Control and Prevention.

The 128 MTB isolates were collected from FQ resistance surveillance sites across China (specifically from Xinjiang, Hebei, and Hunan provinces) and originated from culture-positive TB patients. Following nucleic acid extraction and whole-genome sequencing, all isolates were confirmed to belong to the MTB complex. Based on genotypic profiling of FQ resistance-associated genes, 50 isolates were classified as FQ-susceptible, and 78 were identified as FQ-resistant.

**FIGURE 2:**
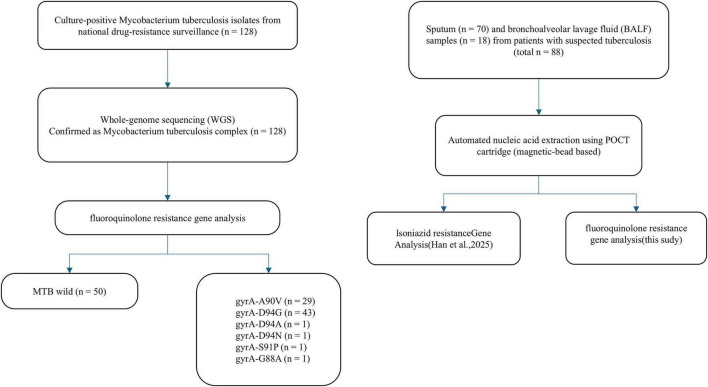
Flowchart of the study design and sample selection. The study comprised two evaluation phases: (Left) Analytical performance evaluation using 128 MTB clinical isolates. These isolates were characterized by Whole Genome Sequencing (WGS) and classified into fluoroquinolone-susceptible (wild-type, *n* = 50) and resistant (mutant, *n* = 78) groups based on *gyrA* gene analysis. (Right) Clinical validation using 88 respiratory specimens (70 sputum and 18 bronchoalveolar lavage fluid) from patients with suspected tuberculosis. These samples were processed using the automated POCT-DO-RAP system to evaluate its diagnostic performance in a clinical setting.

Additionally, 88 archived clinical specimens from patients with suspected tuberculosis were collected from Hebei Provincial People’s Hospital, including 70 sputum samples and 18 bronchoalveolar lavage fluid (BALF) samples. These specimens were used to evaluate the clinical performance of the POCT-DO-RAP assay. The sample selection criteria and overall study workflow are illustrated in Figure 2.

### 2.2 Design and synthesis of probes and primers

The *gyrA* gene sequence (Gene ID: 887105) was retrieved from the NCBI GenBank database and used as the reference for primer and probe design. Oligo 7 software was employed to design candidate primers and probes for the RAA system, with primer lengths set at approximately 30 bp to minimize primer–dimer formation and to ensure an amplicon size of 100–200 bp. The specificity of all designed primers and probes was evaluated using the Primer-BLAST tool available on the NCBI website.

To enhance single-nucleotide discrimination, locked nucleic acid (LNA) modifications were introduced at the mutation sites of the *gyrA* probes. Primers used for qPCR and nested PCR were designed based on previously published literature (Singhal et al., 2016). All primers and probes were synthesized by Bioligo Biotechnology (Shanghai) Co., Ltd., and their detailed sequences are listed in Table 1.

**TABLE 1:** Primer and probe information.

Gene	Primer/probe	Sequence (5′–3′)	Source
*gyrA*	*gyrA*-RAP-F	CCGAGACCATGGGCAACTACCACCCGCAC	This study
	*gyrA*-RAP-R	GGTGGGTCATTGCCTGGCGAGCCGAAGTT	This study
	*gyrA*RS-F	CAGCTACATCGACTATGCGA	(Singhal et al., 2016)
	*gyrA*PRR-R	ATTTCCCTCAGCATCTCCA	(Singhal et al., 2016)
	*gyrA*PRR-F	GACTATGCGATGAGCGTGAT	(Singhal et al., 2016)
	*gyrA*RS-R	GGGCTTCGGTGTACCTCAT	(Singhal et al., 2016)
	*gyrA*90-WT-p	VIC-ACGCGTCGATCTAC	This study
	*gyrA*94-WT-p	FAM-CTACGACAGCCTGG	This study
	*gyrA*-A90V-p	VIC- ACGTGTCGATCTAC	This study
	*gyrA*-D94G-p	FAM- CTACGGCAGCCTGG	This study
	*IS1081*-RAP-F	TGCAGAACCCACTACGCAGCCAATCTGATG	This study
	*IS1081*-RAP-R	ACCCGATCATATTGGGCAACAACTGATTCG	This study
	*IS1081*-p	CY5- CAGCCACCCCGAAGCCCTCCT	This study

The underline indicates the position of the LNA.

### 2.3 Preparation of recombinant plasmids and standard strains

The target sequence within the *gyrA* promoter region was cloned into the pUC57 vector to construct a recombinant plasmid. Plasmid synthesis and assembly were performed by Qingke Biotechnology Co., Ltd. The plasmid DNA concentration was quantified using the Qubit dsDNA High-Range Assay Kit together with a Qubit 2.0 fluorometer. The plasmid copy number per microliter was calculated according to the following equation: plasmid concentration (copy number/μL) = [6.02 × 1,023 × concentration (ng/μL) × 10−9]/ [plasmid size × 660]

A 10-fold serial dilution of the recombinant plasmid was then prepared in 1 × TE buffer to generate a standard range from 105 to 10^0^ copies/μL, and aliquots were stored at –80°C for future use.

To simulate bacterial loads similar to those encountered in clinical samples, the reference MTB strain H37Rv was cultured and subsequently subjected to 10-fold serial dilution in 1 × TE buffer, yielding bacterial suspensions ranging from 104 to 10^1^ CFU/mL. All diluted bacterial standards were stored at –80°C until use.

### 2.4 Establishment and optimization of the integrated POCT-DO-RAP workflow

The POCT-DO-RAP workflow was established based on our previous study (Han et al., 2025) and optimized using the commercially available real-time PCR based POCT device C10 (TargetingOne Corporation Ltd., Beijing, China). The cartridge was designed based on a magnetic bead–based nucleic acid extraction strategy and contains seven chambers, including lysis buffer, magnetic bead mixture, two wash buffers, waste reservoir, elution buffer, and mineral oil. In addition, one sample chamber and two PCR reaction tubes (WT and MT) are included (as illustrated in the schematic). The extraction procedure comprises four automated steps: lysis, magnetic bead binding, washing, and elution. Lysis and binding times were systematically adjusted under different conditions to achieve optimal nucleic acid recovery.

Following nucleic acid extraction, the eluted DNA is automatically dispensed into the WT and MT reaction tubes. The POCT device C10 includes an integrated mechanical module capable of performing autonomous pipette mixing throughout the extraction process, as well as dedicated temperature-control and magnetic-rack units to ensure efficient lysis, binding, washing, and elution. After extraction is completed, the system transfers the eluted nucleic acid into the corresponding PCR tubes. In the final preparation step of the amplification reaction, the instrument aspirates Mg^2+^ solution from an independent magnesium reservoir and delivers it into each PCR tube, followed by automated sealing with mineral oil. Subsequently, the robotic arm closes the tube caps and transfers the reaction tubes to the PCR amplification module. The optimized thermal cycling protocol consisted of an initial RAA phase at 40°C for 10 min, followed by a PCR phase: pre-denaturation at 95°C for 30 s, and 30 cycles of denaturation at 95°C for 10 s and annealing/extension at 62°C for 30 s.

Because the only difference between WT and MT tubes lies in the probe sequences, optimization experiments were performed solely with the WT tube. To enhance analytical specificity and sensitivity, betaine was included in the reaction mixture to reduce secondary structure formation (Kang et al., 2005), and its optimal concentration was determined using 10 copies/μL plasmid templates. Under the same template concentration, different Mg^2+^ concentrations (8, 9, 10 mM) were evaluated to identify the optimal POCT-DO-RAP reaction conditions.

Quality Control: To prevent false-negative results due to PCR inhibition or extraction failure, the conserved insertion sequence *IS1081* was detected in every reaction as an endogenous Internal Control (IC). For external quality assessment, a positive control (H37Rv genomic DNA) and a negative control (sterile water) were tested periodically (e.g., daily or prior to each new reagent batch) to verify system stability, rather than running them simultaneously with every clinical sample due to the limited throughput of the portable device.

### 2.5 Analytical sensitivity and specificity of the POCT-DO-RAP assay

To evaluate the analytical sensitivity of the optimized POCT-DO-RAP assay in comparison with conventional qPCR, recombinant plasmids containing either wild-type or mutant *gyrA* sequences (*gyrA*90-WT, *gyrA*94-WT, A90V, and D94G) were tested across a dilution range of 1–105 copies/μL. To assess the overall sensitivity of the integrated POCT workflow—including sample preprocessing and nucleic acid extraction—MTB reference strain H37Rv was spiked into sputum from healthy donors at concentrations of 10–104 CFU/mL to generate simulated clinical samples. A sputum liquefaction step was included prior to DNA extraction. Nuclease-free water was used as the negative control in all experiments. Each simulated sample series contained an internal control, and the assay sensitivity and reproducibility were evaluated by performing eight independent runs on different days. The optimized DO-RAP reaction conditions and workflow are described in Section 3.2.

Conventional qPCR was performed using the PN101 kit (Vazyme, Nanjing, China) in a total reaction volume of 20 μL, consisting of 4 μL of 5 × Taq Pro buffer, 0.2 μL of Taq Pro HS DNA polymerase (5 U/μL), 0.4 μL each of the forward and reverse *gyrA* primers (10 μM), 0.2 μL each of the gyrA-A90V-p-WT or -p-MT probes (10 μM), 0.2 μL each of the *gyrA*-D94G-p-WT or -p-MT probes (10 μM), 12.6 μL nuclease-free water, and 2 μL of DNA template. The qPCR cycling conditions were as follows: 95°C for 2 min, followed by 40 cycles of 95°C for 15 s and 52°C for 30 s with fluorescence acquisition.

The analytical specificity of the optimized POCT-DO-RAP assay was assessed using five common respiratory bacterial species (*Haemophilus influenzae*, *Streptococcus pneumoniae*, *Staphylococcus aureus*, *Klebsiella pneumoniae*, and *Pseudomonas aeruginosa*) as well as 128 clinical MTB isolates. All MTB isolates were genotypically characterized for FQ susceptibility or resistance by Sanger sequencing at the National Institute for Communicable Disease Control and Prevention, Chinese Center for Disease Control and Prevention.

### 2.6 Sensitivity of the POCT-DO-RAP assay for detecting heteroresistance

To assess the capability of the POCT-DO-RAP assay in detecting heteroresistance, recombinant plasmids containing both wild-type and mutant *gyrA* sequences (*gyrA*-D94G and *gyrA*-A90V) were mixed at defined proportions.

For each reaction, the total plasmid concentration was adjusted to 10^2^ copies/μL.

Mutant and wild-type templates were combined to generate mixtures containing 0, 1, 5, 20, 50, and 80% mutant alleles, and each mixture was tested using the MT tube. This design enabled evaluation of the assay’s ability to identify low-abundance mutant alleles within mixed genetic backgrounds and determine the detection limit for heteroresistance under low template conditions.

### 2.7 Clinical performance of the POCT-DO-RAP assay

A total of 88 clinical specimens were used to evaluate the clinical performance of the POCT-DO-RAP assay for detecting MTB drug resistance. The results obtained by POCT-DO-RAP assay were compared with those from conventional qPCR. The discrepant results were resolved by nested PCR followed by Sanger sequencing.

Nested PCR was performed in two rounds. In the first round, primers *gyrA* RS-F and *gyrA* PRR-R were used; in the second round, primers *gyrA* PRR-F and *gyrA* RS-R were employed (primer sequences listed in Table 1). Nested PCR was conducted according to the instructions of the PN101 kit. Both rounds of amplification were carried out under identical conditions: initial denaturation at 95°C for 2 min, followed by 45 cycles of 95°C for 15 s, 60°C for 30 s, and 72°C for 30 s. Nuclease-free water was included as a negative control in each round. The nested PCR products were sent to Sangon Biotech (Shanghai, China) for Sanger sequencing.

Statistical analyses were performed using IBM SPSS Statistics 21 (IBM, United States). Agreement between different detection methods was assessed using the Kappa statistic.

## 3 Results

### 3.1 Establishment and optimization of the integrated POCT-DO-RAP workflow

The POCT-DO-RAP workflow was systematically optimized using the MTB reference strain H37Rv as the template. The finalized nucleic acid extraction protocol required approximately 30 min from the onset of lysis. The optimized extraction system consisted of 200 μL of liquefied sputum or BALF sample, 400 μL of lysis buffer, 300 μL of magnetic bead suspension, two washing steps with 400 μL wash buffer each, 100 μL of elution buffer, and 50 μL of mineral oil.

Following extraction, 2 μL of the eluted nucleic acid was automatically dispensed into both the WT and MT PCR tubes. The system then aspirated 2 μL of Mg^2+^ solution from an independent reservoir and added it to each reaction tube. Subsequently, 20 μL of mineral oil was overlaid to seal the reaction chambers. The robotic arm then closed the reaction tube caps and transferred the tubes into the PCR module to initiate downstream amplification.

To achieve optimal amplification performance, the effects of betaine concentration and Mg^2+^ concentration on amplification efficiency and fluorescence signal stability were systematically evaluated.

Optimisation experiments revealed that both components exerted significant influence on the DO-RAP reaction. For betaine, a concentration of 0.4 M produced the most stable and robust amplification curves (Supplementary Figure 1A). Reducing the concentration to 0.2 M resulted in insufficient amplification efficiency, whereas increasing it to 0.6 M caused attenuated or distorted fluorescence signals. These adverse effects at higher concentrations are likely due to excessive lowering of DNA melting temperature, impaired DNA polymerase activity, disruption of recombinase–SSB–DNA complex formation during the RAA phase, altered ionic strength, and increased primer–dimer or nonspecific annealing events.

Similarly, Mg^2+^ concentration had a pronounced impact on amplification performance. A final concentration of 9 mM provided the optimal balance between polymerase activity and primer–template specificity (Supplementary Figure 1B). At 8 mM, insufficient Mg^2+^ limited polymerase activity and weakened primer annealing stability, resulting in delayed Ct values. Conversely, at 10 mM, excess Mg^2+^ may stabilize nonspecific secondary structures or compromise polymerase fidelity, leading to reduced fluorescence intensity and elevated background noise.

Collectively, these results demonstrate that 0.4 M betaine and 9 mM Mg^2+^ constitute the optimal reaction conditions for the POCT-DO-RAP assay.

Based on these optimization experiments, the final WT-tube POCT-DO-RAP reaction mixture (20 μL total volume) consisted of: 8 μL RAA reaction buffer and lyophilized enzyme mix, 0.2 μL PN101 DNA Taq polymerase (5 U/μL), 0.6 μL *gyrA*-F (10 μM), 0.6 μL *gyrA*-R (10 μM), 0.2 μL *gyrA*90-WT-p (10 μM), 0.2 μL *gyrA*94-WT-p (10 μM), 0.6 μL *IS1081*-F (10 μM), 0.6 μL *IS1081*-R (10 μM),0.1 μL *IS1081*-p (10 μM), 1.6 μL betaine (4 M), and 3.3 μL nuclease-free water. Following nucleic acid extraction, the instrument automatically added 2 μL of 90 mM Mg^2+^ solution (yielding a final Mg^2+^ concentration of 9 mM), along with 2 μL of the extracted nucleic acid template.

The amplification protocol was as follows: an initial RAA phase at 40°C for 10 min, followed by a PCR phase consisting of 95°C for 30 s for pre-denaturation, and 30 cycles of 95°C for 10 s (denaturation) and 62°C for 30 s (annealing/extension).

### 3.2 Analytical sensitivity and specificity of the POCT-DO-RAP assay

**FIGURE 3:**
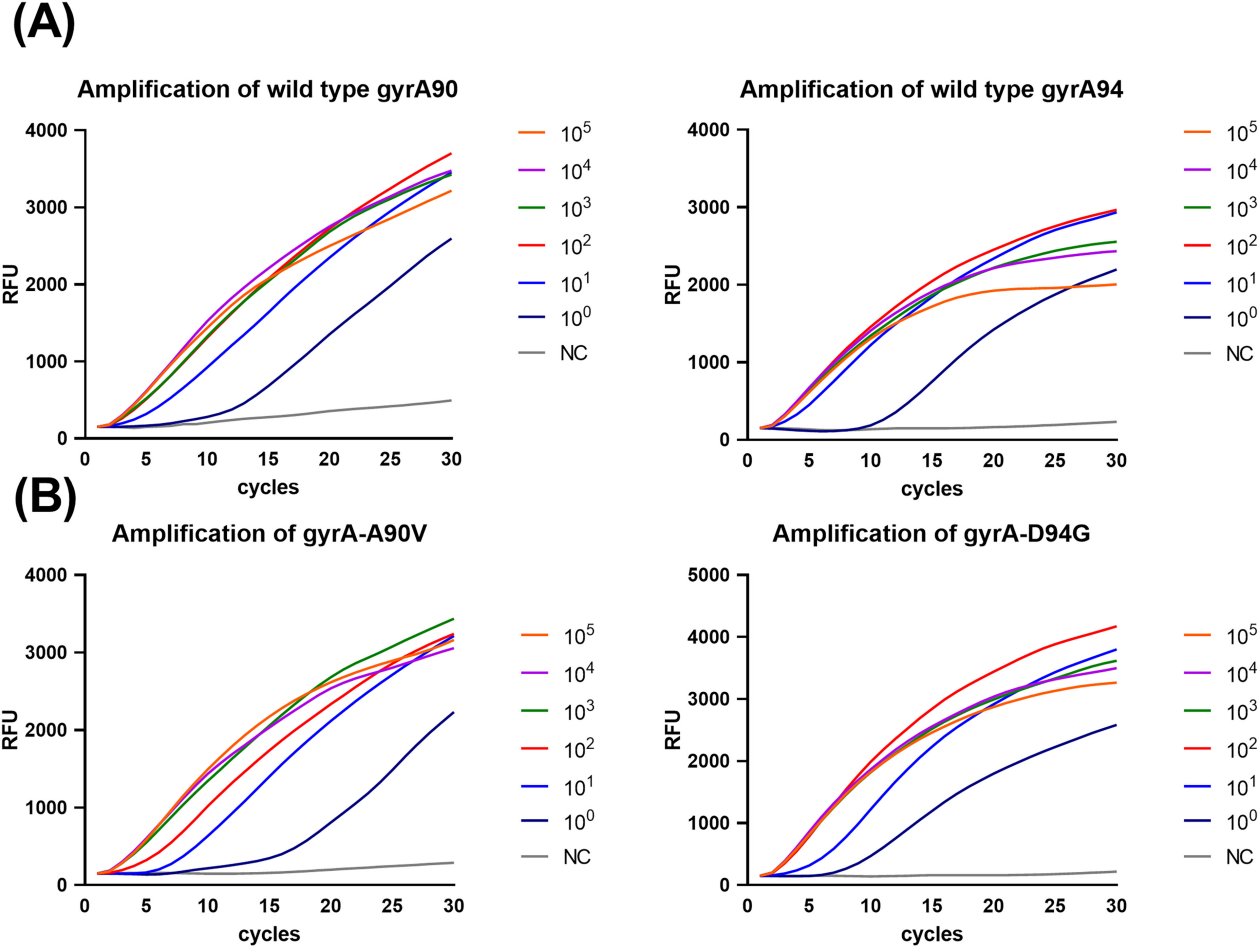
Analytical sensitivity of the POCT-DO-RAP assay using serial plasmid dilutions. **(A)** Amplification curves of WT (*gyrA*90 and *gyrA*94) tube detecting 1–10^5^ copies/μL wild-type plasmid. **(B)** Amplification curves of MT (*gyrA*-A90V and *gyrA*-D94G) tube detecting 1–10^5^ copies/μL mutant plasmid.

**FIGURE 4:**
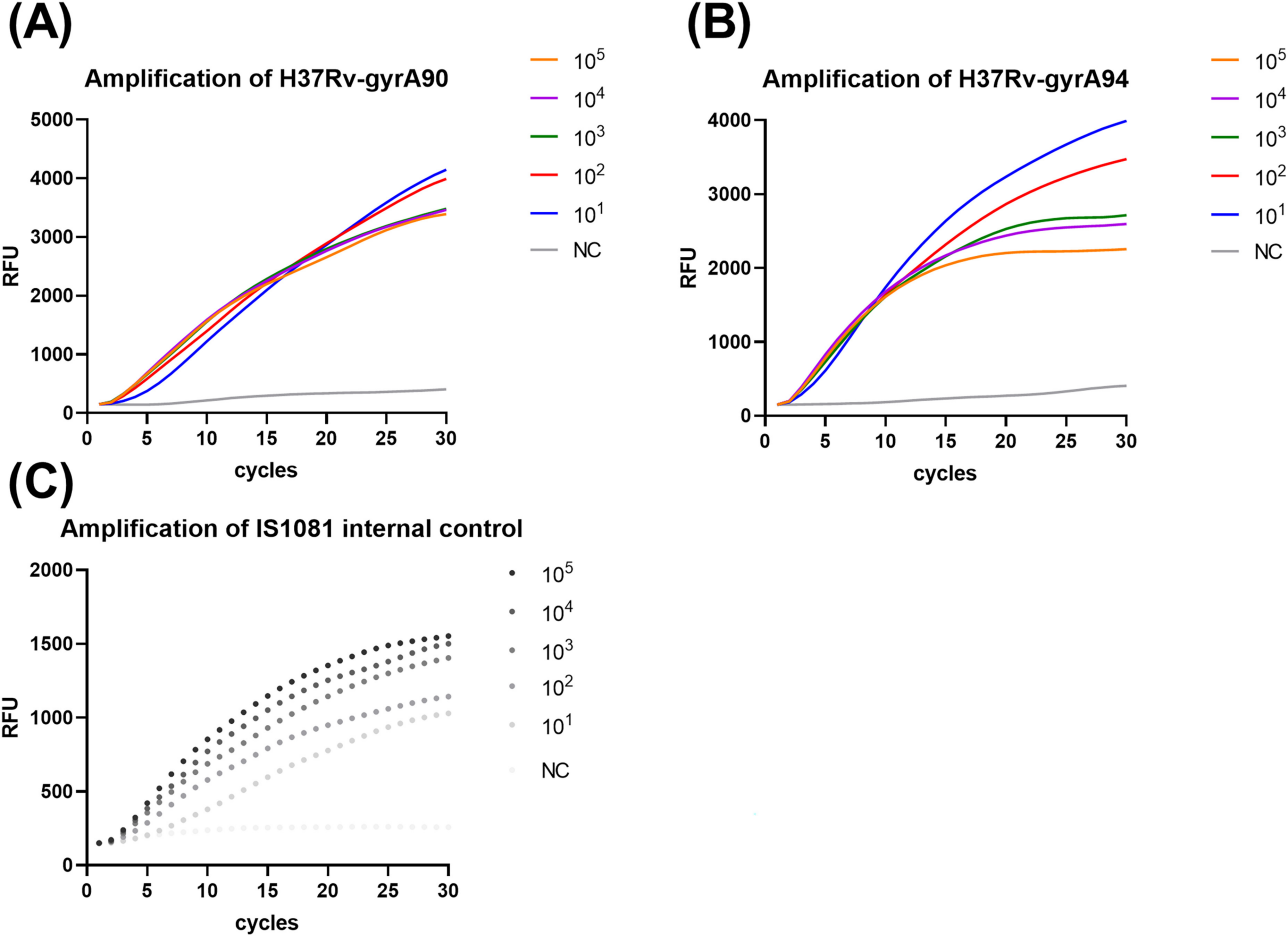
Sensitivity of the POCT-DO-RAP assay for detecting simulated sputum samples. **(A)** Amplification curves of H37Rv-*gyrA*90. **(B)** Amplification curves of H37Rv-*gyrA*94. **(C)** Amplification curves of the internal control *IS1081*.The assay was evaluated using sputum samples spiked with H37Rv concentrations ranging from 10 to 10^5^ CFU/mL.

The analytical sensitivity of the POCT-DO-RAP assay was evaluated using serially diluted recombinant plasmids and H37Rv-spiked healthy sputum specimens. The results demonstrated that the limit of detection (LOD) for both the WT and MT reaction tubes was 1 copy per reaction (Figure 3) and 10 CFU/mL (Figure 4). In comparison, the LOD of qPCR was 10 copies per reaction (Supplementary Figure 2A) and 100 CFU/mL (Supplementary Figure 2B), indicating that POCT-DO-RAP achieved approximately 10-fold higher sensitivity than conventional qPCR.

The specificity assessment showed that all 50 wild-type MTB isolates generated fluorescence signals exclusively in the WT tube. Among the mutant strains, *gyrA*-A90V (*n* = 29), *gyrA*-D94G (*n* = 43), and dual-mutation isolates (*n* = 2) produced distinct fluorescence signals in their corresponding mutation-specific tubes. The DO-RAP results were fully concordant with Sanger sequencing. For other mutation types without corresponding mutation-specific probes (*gyrA*-D94A, *gyrA*-D94N, *gyrA*-S91P, *gyrA*-G88A), only WT-tube fluorescence was observed, consistent with their probe-design characteristics. These results collectively demonstrate the high specificity of DO-RAP for distinguishing wild-type and mutant alleles.

Furthermore, no cross-reactivity was detected with any of the five common respiratory bacterial species tested—*Haemophilus influenzae*, *Streptococcus pneumoniae*, *Staphylococcus aureus*, *Klebsiella pneumoniae*, and *Pseudomonas aeruginosa*—all of which yielded negative results (Table 2). This confirms that the POCT-DO-RAP assay possesses excellent target specificity and does not cross-react with non-mycobacterial respiratory pathogens.

**TABLE 2:** Specificity of the POCT-DO-RAP.

Isolated strain	Number	Result of WT tube		Result of MT tube	
		Wild type of *gyrA*90	Wild type of *gyrA*94	*gyrA*-A90V	*gyrA*-D94G
MTB wild	50	Positive	Positive	Negative	Negative
MTB *gyrA*-A90V	29	Negative	Positive	Positive	Negative
MTB *gyrA*-D94G	43	Positive	Negative	Negative	Positive
MTB *gyrA*-A90V, *gyrA*- D94G	2	Negative	Negative	Positive	Positive
*gyrA*-D94A	1	Negative	Positive	Negative	Negative
*gyrA*-D94N	1	Negative	Positive	Negative	Negative
*gyrA*-S91P	1	Positive	Negative	Negative	Negative
*gyrA*-G88A	1	Positive	Negative	Negative	Negative
*H. influenzae*	1	Negative	Negative	Negative	Negative
*S. pneumoniae*	1	Negative	Negative	Negative	Negative
*S. aureus*	1	Negative	Negative	Negative	Negative
*K. pneumoniae*	1	Negative	Negative	Negative	Negative
*P. aeruginosa*	1	Negative	Negative	Negative	Negative

### 3.3 Sensitivity of the POCT-DO-RAP assay for detecting FQ heteroresistance

To determine the ability of the POCT-DO-RAP assay to detect FQ heteroresistance, mixtures of recombinant plasmids containing wild-type and mutant *gyrA* sequences were prepared at total template concentrations of 100 copies per reaction.

Across mutant proportions ranging from 1 to 80%, the MT tube consistently generated positive amplification signals for the mutant allele.

The assay successfully detected mutant DNA present at as low as 1% within a predominantly wild-type background (Supplementary Figure 3), demonstrating its high analytical sensitivity for identifying low-frequency resistant subpopulations. These results indicate that POCT-DO-RAP is capable of reliably detecting clinically relevant heteroresistance even when the mutant population is present at very low abundance.

### 3.4 Clinical performance of the POCT-DO-RAP assay

A total of 88 clinical sputum specimens were tested using both the POCT-DO-RAP assay and conventional qPCR. According to the qPCR results, 41 samples were classified as susceptible (positive), 5 yielded borderline results (CT > 38), and 42 were negative; no resistant isolates were detected. In comparison, POCT-DO-RAP identified 50 samples as susceptible and 38 as negative, also without detecting any resistant isolates. Notably, 9 samples that were categorized as borderline or negative by qPCR were confirmed as positive by both DO-RAP assay and nested PCR followed by Sanger sequencing, indicating a superior detection capability of the POCT-DO-RAP assay for low-abundance targets.

Using nested PCR followed by Sanger sequencing as the reference standard, the sensitivity, specificity, positive predictive value (PPV), negative predictive value (NPV), and Cohen’s kappa coefficient for qPCR were 82, 100, 100, 80.9, and 0.797%, respectively. In contrast, the POCT-DO-RAP assay achieved 100% sensitivity, specificity, PPV, and NPV, with a kappa value of 1.0 (Table 3), demonstrating perfect agreement with the reference method and superior clinical performance over conventional qPCR.

**TABLE 3:** Clinical performance of POCT-DO-RAP in 88 samples compared with Sanger sequencing.

Method	Wild	Mutation	Negative	Total	Sensitivity (%)	Specificity (%)	PPV (%)	NPV (%)	Kappa
Sanger sequencing	50	0	38	88					
qPCR	41	0	47	88	82.00	100.00	100.00	80.9	0.797
POCT-DO-RAP	50	0	38	88	100.00	100.00	100.00	100	1.000

## 4 Discussion

FQs play a pivotal role in the treatment of MDR/RR-TB (Collaborative Group for the Meta-Analysis of Individual Patient Data in Mdr-Tb treatment–2017; Ahmad et al., 2018), and therapeutic outcomes are strongly influenced by mutations within the *gyrA* quinolone-resistance–determining region (QRDR) (Tu et al., 2025). However, the global burden of FQ resistance is rising, with recent surveillance data indicating that approximately 20% of MDR-TB cases exhibit additional resistance to fluoroquinolones (pre-XDR), creating a severe bottleneck for effective regimen construction. Failure to rapidly identify FQ resistance prior to treatment initiation substantially reduces cure rates and increases the risk of resistant strain transmission (Willby et al., 2020). Therefore, developing a rapid, sensitive, and mutation-resolving diagnostic method that can be deployed in primary healthcare settings is of significant clinical importance. In this study, a POCT-DO-RAP assay was established that integrates automated nucleic acid extraction, dual-stage amplification, and real-time detection within a single device, enabling rapid and user-friendly identification of FQ resistance and providing a promising diagnostic approach for on-site MDR/RR-TB management.

Several widely used molecular diagnostics still exhibit intrinsic limitations. Line probe assays (LPAs) detect major resistance-associated mutations but require smear-positive or culture-positive samples, restricting their applicability in paucibacillary cases (Danchuk et al., 2024; Kania et al., 2025). Xpert MTB/RIF and MTB/XDR offer ease of operation and high automation but have limited capacity to detect low-frequency mutations, typically requiring ≥ 60% mutant DNA to indicate resistance (Cao et al., 2021; Ngabonziza et al., 2020). MeltPro melting-curve assays can cover multiple mutations simultaneously, yet they rely heavily on specialized instrumentation, and data interpretation can be complex (Chang et al., 2025; Hu et al., 2023). Whole-genome sequencing (WGS) provides the most comprehensive resistance information; however, its high cost and technical demands hinder widespread implementation in resource-limited regions (Hu et al., 2024; Wasswa et al., 2022). Conventional qPCR remains simple and broadly applicable but often fails to detect low-abundance bacilli or minor heteroresistant subpopulations (Bustin et al., 2022; Poh et al., 2020). Collectively, these limitations delay early resistance identification and impact timely therapeutic adjustment.

Compared with existing technologies, the POCT-DO-RAP assay developed in this study demonstrates several advantages. First, DO-RAP adopts a stepwise amplification strategy that combines the rapid strand-exchange capability of recombinase-aided amplification (RAA) with the high specificity of qPCR. This dual-stage design markedly enhances template abundance prior to qPCR detection, enabling an analytical sensitivity of 1 copy/reaction or 10 CFU/mL—approximately a 10-fold improvement over conventional qPCR. Notably, all 9 samples that yielded negative or gray-zone qPCR results (CT > 38) were correctly identified by POCT-DO-RAP and subsequently verified by sequencing, underscoring the clear performance advantage for low-bacillary-load specimens.

Second, the POCT device implemented in this study achieves a fully integrated workflow, including automated magnetic-bead extraction, robotic transfer of reagents, Mg^2+^ addition, and in-tube oil sealing, thereby minimizing manual intervention and preventing aerosol contamination. The entire process—from raw specimen to final readout—can be completed within approximately 60 min. These features make the POCT-DO-RAP assay highly suitable for near-patient testing in primary or decentralized healthcare settings, fitting seamlessly into the TB diagnostic algorithm as a rapid triage tool to screen for FQ resistance before referral for comprehensive phenotypic DST.

Third, the POCT-DO-RAP assay achieved excellent single-nucleotide discrimination by incorporating LNA-modified probes. Importantly, POCT-DO-RAP reliably detected mutant alleles even when they represented only 1% of mixed templates, significantly outperforming existing commercial assays such as Xpert. Given that heteroresistance often precedes fixed resistance, the ability to detect low-level mutant subpopulations offers substantial advantages for early clinical decision-making and preventing treatment failure due to expansion of resistant clones.

This study has several limitations. First, the current assay targets only two major *gyrA* mutation sites (A90V and D94G) and does not yet cover other QRDR mutations within *gyrA* or *gyrB*. Although these two mutations account for the vast majority (Xu et al., 2025) of FQ resistance in China and align with high-confidence mutations in the WHO catalog, a negative result by this assay does not entirely rule out resistance caused by rare or non-canonical mutations. Therefore, in clinical practice, reports should include a disclaimer recommending phenotypic drug susceptibility testing (DST) for high-risk patients who test negative for these specific mutations. Future studies may expand mutation coverage through additional primer/probe sets or leverage microfluidic integration to enable high-throughput, parallel detection (Hong et al., 2022; Wu et al., 2024). Second, the number of clinical specimens analyzed in this study remains relatively limited. In the clinical validation phase involving 88 respiratory samples, no FQ-resistant cases were detected. This is likely due to the relatively low prevalence of primary FQ resistance in the general patient population sampled and the limited sample size of MTB-positive cases in this pilot study. However, the assay’s ability to detect FQ resistance mutations was fully validated in the analytical evaluation using 128 genotyped clinical isolates. Therefore, multicenter, large-scale validation is required to further assess diagnostic performance and reproducibility. Additionally, although the current two-tube configuration enables clear discrimination between wild-type and mutant alleles, extending the assay to multiple targets will require further optimization to minimize primer–dimer formation and nonspecific amplification events in multiplex settings.

Compared to other emerging isothermal amplification methods that often lack single-nucleotide specificity, and unlike commercial PCR assays (e.g., Xpert MTB/XDR) that may miss low-abundance heteroresistance, our study bridges a critical gap. The POCT-DO-RAP assay represents a significant advancement by combining three key advantages: (1) ultra-high sensitivity (1 copy/reaction) driven by the dual-stage amplification; (2) superior specificity for single-base mutations enabled by LNA probes, allowing the detection of 1% h e teroresistance; and (3) a fully automated “sample-in, result-out” workflow. These features collectively position it as a promising tool for early identification of FQ-resistant M. tuberculosis, particularly in primary healthcare facilities and resource-limited regions, where it may contribute meaningfully to the timely management and precision treatment of MDR/RR-TB.

## Data Availability

The original contributions presented in this study are included in this article/Supplementary material, further inquiries can be directed to the corresponding authors.
