# Supplementary material for: Development of a point-of-care dual one-step recombinase-aided PCR assay for rapid identification of Mycobacterium tuberculosis gyrA mutations conferring fluoroquinolone resistance
Source: Front Microbiol. 2026 Mar 2;17:1772984. doi: 10.3389/fmicb.2026.1772984 (PMC12989489; doi:10.3389/fmicb.2026.1772984)
Supplement: Supplementary file 1 [file Table_1.docx]

Supplementary Material

## Supplementary Figures

**
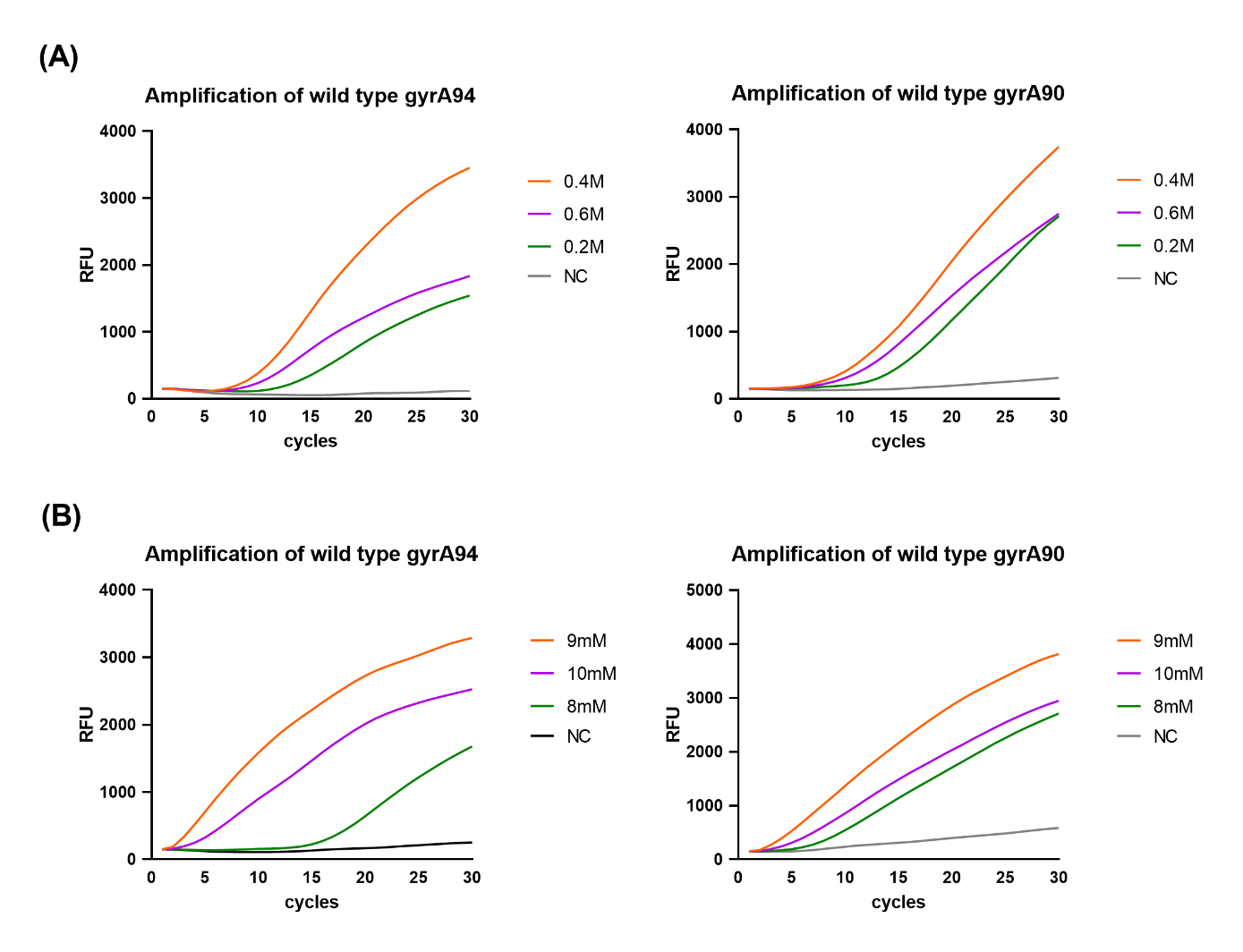
**

**Supplementary Figure 1** Optimization of betaine and Mg²⁺ concentrations for the POCT-DO-RAP assay: (A) Amplification curves of wild-type gyrA90 and gyrA94 targets under different betaine concentrations using a 10¹ copies/μL plasmid template. (B) Amplification performance of wild-type gyrA90 and gyrA94 targets under varying Mg²⁺ concentrations with a 10¹ copies/μL plasmid template.


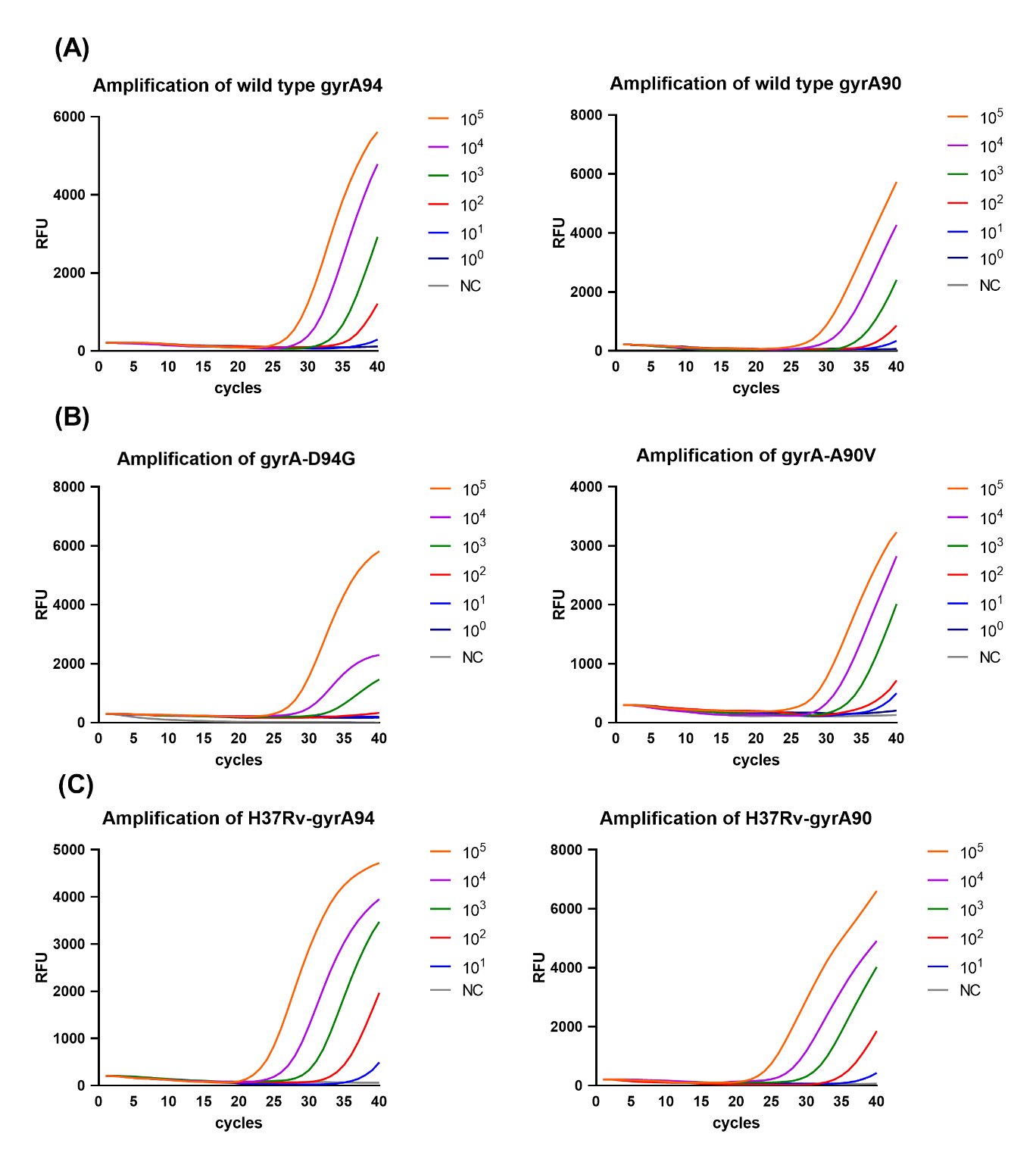


**Supplementary Figure 2** qPCR sensitivity analysis using plasmid dilutions and H37Rv-spiked simulated samples: (A) qPCR amplification curves of wild-type gyrA90 and gyrA94 plasmids across a 1-10⁵ copies/reaction dilution series. (B) qPCR amplification curves of gyrA90-A90V and gyrA94-D94G plasmids across a 1-10⁵ copies/reaction dilution series. (C) qPCR amplification curves of simulated samples prepared by spiking H37Rv standard strain into sputum, ranging from 10 to 10⁵ CFU/mL, targeting wild-type gyrA90 and gyrA94.


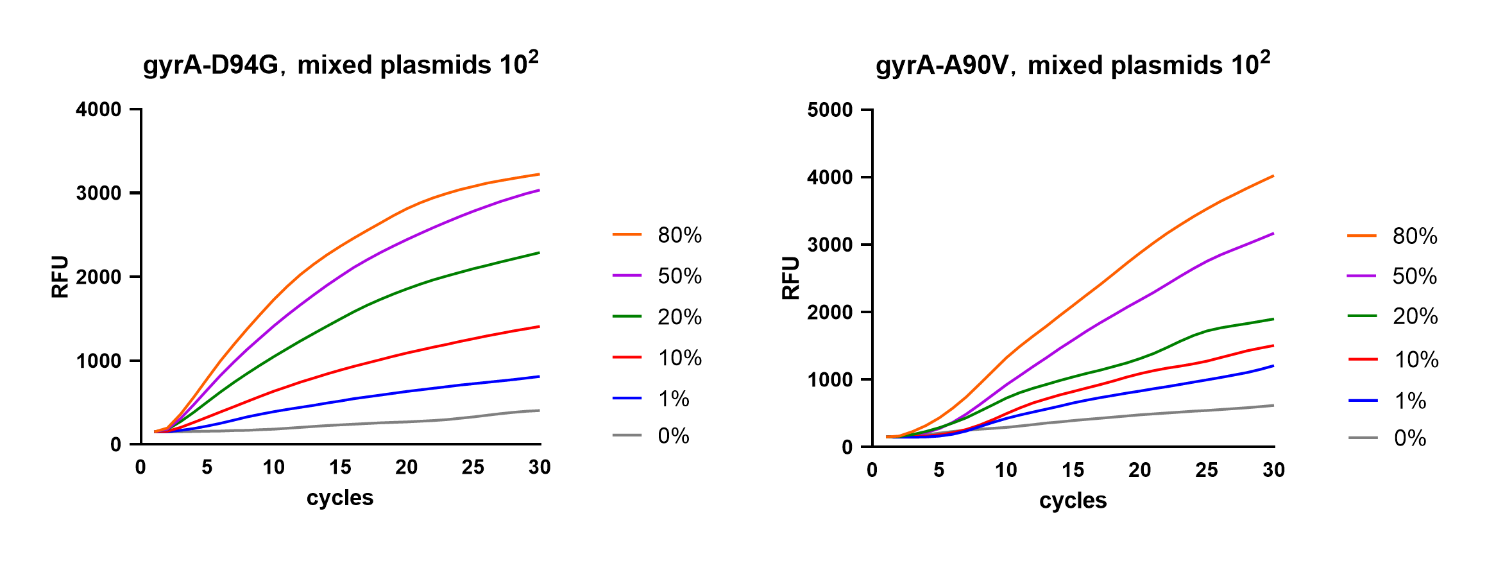
 **Supplementary Figure 3** Evaluation of the POCT-DO-RAP assay sensitivity for detecting gyrA heteroresistance. The figure presents amplification curves from Mutant-Type (MT) tubes for both gyrA D94G and gyrA-A90V mutations. To simulate clinical heteroresistance, mutant plasmids were mixed with wild-type plasmids at varying proportions ranging from 80% down to 1%, while maintaining a fixed total template concentration of 10^2^ copies/μL. The assay demonstrated reliable detection of mutant alleles even at a 1% abundance for both mutation types.
